# Curriculum Co-creation in a Postdigital World: Advancing Networked Learning and Engagement

**DOI:** 10.1007/s42438-022-00304-5

**Published:** 2022-04-01

**Authors:** Tanya Lubicz-Nawrocka, John Owen

**Affiliations:** 1 University of Edinburgh (Edinburgh Global), Edinburgh, Scotland; 2 University of Manchester (School of Health Sciences), Manchester, England

**Keywords:** Curriculum co-creation, Student engagement, Students as partners, Postdigital education, Networked learning

## Abstract

Literature on curriculum co-creation tends to focus on in-person experiences of teaching and learning. However, the Covid-19 pandemic has spurred on learners and teachers to co-create curricula in new and creative ways. This article examines curriculum co-creation in a postdigital world focusing on the connections between curriculum co-creation and networked learning. Drawing on Hodgson and McConnell’s conceptualisation of six key practices of networked learning, the authors explore how these practices connect to curriculum co-creation in theory and in a specific example from a fully online module that ran effectively during the Covid-19 pandemic. The authors conclude that networked learning and curriculum co-creation foster postdigital thinking and dialogue, which advance many elements of excellent learning and teaching to benefit both students and staff as we continue to navigate the ‘new normal’.

## Introduction

The Covid-19 pandemic has resulted in a destabilising period when many teachers and learners needed to adapt quickly to expanding online spaces and new ways of teaching and learning. Motivation, engagement, and relationships have grown in importance as students and staff navigate increasingly digital and hybrid teaching (Jandrić et al. 2020, 2021; Ntem et al. 2020; Hall 2020). According to Raes (2021: 138), ‘[d]uring synchronous hybrid learning both on-site and remote students are connected and taught synchronously’. Pischetola (2021: 70) adds that this will often ‘involve blurred boundaries and spatiotemporal configurations in a context of radical uncertainty’.

During past two decades, the postdigital education literature, including but not limited to the networked learning literature, has evolved far beyond an initial focus on digital technology and e-learning (Fawns 2019). The literature on curriculum co-creation has also burgeoned, stemming from early research into student-centred learning, student engagement, and principles of good practice in undergraduate education (Chickering and Gamson 1987) to its current focus on student-staff collaborations in curriculum design and enhancement (Bovill and Woolmer 2019). We draw on a frequently cited definition of curriculum co-creation by Bovill et al. (2016: 196) stating: ‘Co-creation of learning and teaching occurs when staff and students work collaboratively with one another to create components of curricula and/or pedagogical approaches.’ Curriculum co-creation, as a ‘values-based implementation of an ongoing, creative, and mutually-beneficial process of staff and students working together to share and negotiate decision-making about aspects of curricula’ (Lubicz-Nawrocka 2020), has potential to benefit both students and staff.

The Covid-19 pandemic has spurred increased discussions about values and aims in higher education (Cureton et al. 2021; Ntem et al. 2020; Jandrić et al. 2020, 2021), focusing on their reconfigurations in online environments. In this article, we explore the potential of curriculum co-creation as a forward-thinking approach that will benefit postdigital learning and teaching, especially in the context of hybrid education. We do this by exploring the synergies between theories of curriculum co-creation and conceptualisations of networked learning.

## Curriculum Co-creation and Working with Students as Partners

Curriculum co-creation and student-staff partnerships promote high levels of student and staff engagement, which often occur through staff inviting students to take more active roles in shared decision-making (Bovill 2020b; Lubicz-Nawrocka 2019b; Matthews et al. 2018). Co-creation critically involves negotiation and collaboration (as opposed to full control by staff or students over curricula), with relationships based on respect, reciprocity, and shared responsibility (Cook-Sather et al. 2014). Shared decision-making also involves negotiating issues of power, so staff and students need to build positive working relationships that serve as foundations of their collaborations (Bovill 2020a; Lubicz-Nawrocka 2019a). Curriculum co-creation draws on critical pedagogy to provide alternative possibilities for partnership-based learning and teaching (Bovill et al. 2009; Peters and Mathias 2018) and ‘knowledge socialism’ that facilitates shared decision-making and the co-creation of knowledge (Peters et al. 2020).

Staff and students involved in curriculum co-creation often engage in creative learning and teaching processes that benefit the student experience (Blau and Shamir-Inbal 2018; Kaur et al. 2019; Lubicz-Nawrocka 2019a; Riddell et al. 2021). This can develop co-creators’ resilience when negotiating the curriculum and navigating challenges collaboratively (Cook-Sather et al. 2014; Lubicz-Nawrocka 2019a). Curriculum co-creation develops strong working relationships between students (Vaughan et al. 2016; Owen et al. 2021) and between students and staff (Blau and Shamir-Inbal 2018; Kaur et al. 2019). This often leads to positive outcomes for both students and staff reflecting on meaningful and rewarding learning and teaching experiences (Dollinger et al. 2018; Kaur et al. 2019; Lubicz-Nawrocka 2019a; Temple Clothier and Matheson 2019).

During the Covid-19 pandemic, individuals engaging in curriculum co-creation and partnership have been well positioned to work creatively and resiliently while adapting to changing lived experiences (Ntem et al. 2020; Riddell et al. 2021). Many continued to draw on shared values that help serve as a strong foundation for partnerships in a wide variety of international higher education contexts under normal circumstances and especially in times of crisis (Ntem et al. 2020). Riddell et al. (2021: 112) describe, for example: ‘The global pandemic encouraged us to re-think traditional modes of design and delivery, and in doing so advanced our thinking about how to deconstruct notions of expertise and authority in productive ways.’

## Curriculum Co-creation and Postdigital Education

Fawns (2019) discusses how techno-centric terms such as ‘digital education’ and ‘e-learning’ are commonly used to differentiate between digital and non-digital forms of education or attribute essential technological properties to the design and practice of teaching and learning. Fawns states that learning takes place inside and outside the classroom, in both the virtual and physical world, and technology is present in almost all educational experiences. Therefore, he suggests that the only meaningful claim about ‘online learning’ is that it requires an Internet connection. Fawns (2019: 132) argues that technology and education are interdependent and iterative, suggesting a ‘postdigital perspective in which all education … takes account of the digital and non-digital, material and social, both in terms of the design [and practice] of educational activities’.

In calling for a postdigital perspective, Fawns (2019: 140) advocates a shift in educational culture rather than language, where ‘design work should go beyond setting tasks and configuring environments, and include possibilities for students to configure and customise their own practices’. In this sense, postdigital education recognises the wider context of learning in teaching including who is (not) involved and the agency of each individual. Fawns, Aiken, and Jones (2019) suggest that online learning is less limited by distance or technology than by the other key factors such as time, policy, infrastructure, and pedagogy. During the Covid-19 pandemic, we saw this play out as universities with established online learning policies and infrastructures were in stronger positions to deal with the challenge of quick transitions to online environments. Fawns et al. (2019) also describe the power of various online pedagogies to foster a strong sense of community.

In our view, successful online programmes are the result of students, teachers and administrators learning to work effectively within and around the constraints of infrastructure and policy. It follows that these collaborators should be supported to develop practices that work for them, both individually and collectively. (Fawns et al. 2019: 296)

Similarly, Jandrić (2019: 276) claims that ‘we have no choice but develop new postdigital forms of collective intelligence’ and suggests using the dialectic of ‘we-think, we-learn, we-act’. Expanding on this trialectic, Jandrić suggests that it is our epistemic necessity to recognise how the influence of all the people in our lives (our intellectual ancestry) has informed our thinking and points towards the ‘need to develop we-thinking in, and for the future’. In we-learn, he acknowledges the need for postdigital cross-fertilisation and not (digital, analog, human or technological) cross-sterilisation. Finally, for we-act, Jandrić (2019) suggests that we need to challenge the current social order of identity politics and take individual and group responsibility for our own actions.

This chimes with the literature on curriculum co-creation. This pedagogy fosters the development of postdigital collective intelligence which, in and of itself, can challenge the status quo of academic hierarchies when students and staff enact shared values to work in partnership (Hill et al. 2016; Bovill 2020b; Lubicz-Nawrocka and Bovill 2021). Curriculum co-creation fosters fruitful yet messy ways of working collaboratively in which students and staff navigate the complexities of academic structures and spaces as they integrate diverse perspectives and expertise to work towards shared aims in learning and teaching. This can occur in similar ways to how postdigital dialogue can represent collective knowledge-making that explores the messy and unpredictable interconnections between and complexities within integrating the digital into everyday practice (Jandrić et al. 2018, 2019).

Knox (in Jandrić et al. 2019: 167) suggests that, through postdigital education, ‘we must also find ways to promote and establish thinking and decision-making as reflective political beings… [to] cultivate the kind of thinking and learning we might associate with a critical citizen of our times’. The curriculum co-creation literature similarly indicates that the processes of collaboration, negotiation, and shared decision-making develop a wide range of skills and attributes that benefit students’ personal and professional development (Cook-Sather et al. 2014; Mercer-Mapstone et al. 2017; Blau and Shamir-Inbal 2018). Furthermore, curriculum co-creation provides opportunities for democratic engagement that motivate students to make a difference beyond their modules and contribute as active citizens to their wider communities (Bergmark and Westman 2016; Lubicz-Nawrocka 2019b).

Postdigital thinking (Jandrić et al. 2018) and dialogue (Jandrić et al. 2019) have become increasingly apparent during the Covid-19 pandemic as new forms of collective intelligence that have rapidly appeared at an unprecedented scale. The pivot to online and hybrid teaching and learning has also destabilised traditional ways of working, creating new spaces for students to work collaboratively with staff in this new territory (Jandrić et al. 2020, 2021; Hall 2020; Riddell et al. 2021).

## Curriculum Co-creation and Networked Learning

Hodgson and McConnell (2019) describe the core aspects of networked learning in the postdigital context to show intricate connections between technology and people who use technology within their own settings, spaces, and practices. They share early definitions and principles of networked learning, including the following definition:

Networked collaborative learning (NCL) is therefore the bringing together of learners via personal computers linked to the Internet, with a focus on them working as a ‘learning community’, sharing resources, knowledge, experience, and responsibility through reciprocal collaborative learning. (McConnell 1998)

This definition also resonates with the later definition (Goodyear et al. 2004) that emphasises how online spaces ‘promote connections: between one learner and other learners, between learners and tutors; between a learning community and its learning resources’.

Similarly, the Networked Learning Editorial Collective (2021) describe networked learning research and practice as three intertwined phenomena: human-interpersonal relationships, technology, and collaborative engagement. In the move from ‘emergency remote teaching’ (Hodges et al. 2020) to networked learning during the Covid-19 pandemic, they suggest an updated definition of networked learning through a postdigital lens, with a strong emphasis on interpersonal connections (Networked Learning Editorial Collective 2021: 320).

Networked learning involves processes of collaborative, co-operative, and collective inquiry, knowledge-creation, and knowledgeable action, underpinned by trusting relationships, motivated by a sense of shared challenge, and enabled by convivial technologies. Networked learning promotes connections: between people; between sites of learning and action; between ideas, resources, and solutions; across time, space, and media. Like Hodgson and McConnell (2019) and the Networked Learning Editorial Collective (2021), we see networked learning as a human-centric form of postdigital education.

As in networked learning, curriculum co-creation is highly contextualised, based on relationships within module and programme learning communities, and it draws on the wide range of expertise of both students and staff as they work in partnership towards shared aims (Bovill 2020a; Burnapp et al. 2018; Kaur et al. 2019). Hodgson and McConnell (2019) highlight how networked learning promotes connections between students and staff as well as their resources and spaces in which they work both online and offline, which resonates with the connections and relationships that are developed through co-creation. Furthermore, both networked learning and curriculum co-creation draw on Freire’s work (1972) to advance critical pedagogy and democratic engagement through the recognition of power dynamics in education (Auerbach 1993; Bovill et al. 2009; Hodgson and McConnell 2019; Peters and Mathias 2018).

Networked learning and curriculum co-creation both promote open discussions of power and privilege while drawing on a foundation of strong working relationships between staff and students to give each a sense of agency and responsibility for the learning community, which can advance social justice. Fostering different ways of working by taking networked learning and co-creation approaches can help us to be more inclusive in curriculum design and enhance learning and teaching.

Hodgson and McConnell describe the following eight principles of networked learning pedagogy:

1. The focus is on learning which has a perceived value to the learners.
2. Responsibility for the learning process should be shared (between all actors in the network).
3. Time has to be allowed to build relationships.
4. Learning is situated and context dependent.
5. Learning is supported by collaborative or group settings.
6. Dialogue and social interaction support the co-construction of knowledge, identity, and learning.
7. Critical reflectivity is an important part of the learning process and knowing.
8. The role of the facilitator/animator is important in networked learning. (Hodgson and McConnell 2019: 45–46)

All eight principles resonate with the co-creation theory and practices. Principle 6 focusing on co-construction of knowledge, identity, and learning bears close similarities with curriculum co-creation. The engaging, student-centred nature of curriculum co-creation often supports students and staff to share responsibility for learning as well as some decisions affecting teaching while reflecting and recognising its benefits (principles 1, 2, and 7) (Blau and Shamir-Inbal 2018; Bovill 2020b; Dollinger et al. 2018; Lubicz-Nawrocka 2018, 2019a). In networked learning, ‘[p]ower, age, gender, identity, socio-cultural norms, language, and discourse are all recognised as important dimensions and influences on the process and experience of taking responsibility’ (Hodgson & McConnell 2019: 46). Likewise, these dimensions of social justice underpin curriculum co-creation practices (Bergmark and Westman 2016; Bovill 2020b; Cook-Sather et al. 2018; Lubicz-Nawrocka 2019b).

Furthermore, curriculum co-creation is an evolving process that necessitates time to build relationships, trust, and reciprocity through dialogic learning and teaching that recognise the nature and context of each learning community (principles 3, 4, 5, and 6) (Bergmark and Westman 2016; Bovill 2020a; Cook-Sather et al. 2014; Lubicz-Nawrocka 2019b). The role of staff—principle 8—is also critical in creating opportunities for curriculum co-creation, which is distinct from either full control over curriculum development by staff (which is typically the case) or full control over curriculum by students (Boomer 1992; Bovill and Bulley 2011; Cook-Sather et al. 2014).

Drawing on the eight principles, Hodgson and McConnell present six key practices that are present in high-quality networked learning:

1. ‘Democracy and openness in the educational process’.
2. ‘Self-determined/managed learning’ that facilitates participants’ ‘freedom to learn’.
3. ‘A real purpose in the cooperative process.’
4. ‘A supportive learning environment.’
5. ‘Collaborative assessment of learning.’
6. ‘Evaluation of the ongoing learning process’ (Hodgson and McConnell 2019: 51–52).

Hodgson and McConnell (2019: 56) further reference student-staff partnership in networked learning pedagogy by stating: ‘Our experience indicates the many learning benefits of involving students as active partners in decision making about the course, and in its ongoing design’.

This statement resonates with us in understanding the connections between networked learning and curriculum co-creation, but we feel there is a gap in the literature bringing together these concepts. We were initially drawn to the six key elements of networked learning through our research and experiences of curriculum co-creation in practice, and we felt a close examination of their synergies would be beneficial. Therefore, in the next sections, we present a recent example from one of the co-author’s teaching, before we delve into discussing each of these six networked learning practices to show how they are manifested in both the example as well as in further curriculum co-creation examples from the literature.

## An Example of Co-created Networked Learning in Practice

This example illustrates how a new module on an established online distance learning programme presented an opportunity to take a more co-created approach than previously taken on the programme. The Masters of Public Health (MPH) is a global online learning programme at The University of Manchester.^1^ It has been running since 2002 and has a diverse student population (approximately 400 students from around 100 countries) with options for studying part-time, full-time or for professional development. The MPH offers a range of modules from the traditional public health subjects such as Epidemiology to more contemporary subjects such as Climate Change and Health.

The Introduction to Public Health module was newly introduced in the 2020–2021 academic year, with 25 students enrolled. The two module assessments discussed here were designed by the module teacher to encourage active engagement and provide opportunities for student co-created learning. The first assessment required students to reflect on their personal motivations for studying, through shared reflective blog posts. The second assessment was designed to engage students in a team-based, applied project that brought together their learning from throughout the module. It was hoped that the collaborative opportunities and increased sense of student responsibility would help form the basis of a learning community for this cohort of students. Both assessments gave students the responsibility to determine their own topics, individually in a blog and collectively in a team project. Although the team project and blog are not uncommon types of assessment in higher education, they are not the norm on the wider MPH programme. Even though the assessments were designed by the module tutor, the students’ freedom on some aspects of both assessments demonstrate an alternative approach to current programme assessments (both in the format and the co-created nature), somewhat challenging the status quo.

John Owen, the module leader, shares feedback from students and his own reflections. This work was considered to not require ethical approval from The University of Manchester. Student feedback was gathered anonymously through several methods: a specific survey about their experience of the blog assessment, the standard end of module survey, and an online feedback form at the end of each topic.

### Blog-based Assessment

The open and personally reflective nature of the blog assessment particularly highlighted its self-determined nature. Students were given the freedom to be creative with their writing and could reflect on any experiences that motivated them to study. They were encouraged to develop memorable or intriguing titles and include relevant images to enhance engagement with their blog posts. The posts uncovered some deeply emotional experiences for some students and, although writing about these on an open platform was daunting for some, the majority expressed their enjoyment in engaging in the process.

For example, one student commented on the diversity of the blog posts, which were ‘all very different but equally inspiring’. Another student described how the blog-based assessment developed their understanding of the course and the backgrounds, experiences, and perspectives of fellow students. The context-dependent and inclusive nature of the assessed blog presented a culturally and personally relevant opportunity for students to co-create and knowledge-make through the resulting blog publication ‘Why Public Health?’ (University of Manchester 2020).

Students embraced the collaborative and supportive learning environment, sharing feedback by commenting on draft blogs that resulted in emerging peer-to-peer bonds early in the module. Students commented on the supportive learning environment. For instance, one student said:

...seeing the wealth of experiences, knowledge and perspectives through the Blogs I can see that we all have incredibly valuable perspectives to bring to the course and I am sure there will be so much that we can learn from each other.

This enhanced sense of community and collaboration supported students to gain agency over their learning, which enhanced their sense of purpose. Another student said:

I think this is an excellent way to start the course. It really helped to crystalise why I am doing it and as I started my first two modules with trepidation this helped me to focus and created the energy I needed to get going.

This student’s sense of ‘energy’ is seen here in developing their confidence and their intrinsic motivation to engage with online learning with a better understanding of how their learning relates to them as an individual and their wider aims.

### Team Project

A team project in the second half of the module built on the aforementioned blog-based co-creation experience and also provided students with real purpose to collaborate in diverse, globally connected teams on an authentic problem. Students negotiated and co-created their projects within their teams, deciding on their project aims and objectives; their team’s specific approach; and the technology they adopted for communication, collaboration, and presentation.

Students were encouraged to discuss the project’s assessment criteria and ask questions to each other and their tutors. Some students specifically commented on the detailed assessment criteria and how the opportunities to discuss these developed their understanding of the requirements of the assessment, suggesting potential for enhancing students’ assessment literacy and meta-cognitive awareness.

Peer assessment criteria were also used for each student to evaluate their peers’ individual contributions within their teams. Teachers actively encouraged students to examine and discuss both the project’s overall assessment criteria and the peer assessment criteria. To help students practice giving peer assessment based on the criteria, an interim peer evaluation part-way through the project provided the students with formative feedback.

Several students commented on their experience of the team project. Some students appreciated the collaborative and creative opportunities of working together in teams. For example, one student reflected on how it was beneficial ‘to have the opportunity to conduct group work and use a bit of creativity and flair instead of just essays, [and] nice to have freedom of subject choice’. Furthermore, another student highlighted how the groupwork enhanced their sense of community, which led to increased engagement with the module: ‘Working in a small team has been fantastic – I feel like I really got to know some fellow students and had so much more engagement with this module than others because we were working together.’

Other students reported an increase in their social presence in the online environment through the collaborative experience: ‘I enjoyed the team project as it allowed us to meet other students, I feel this is important as most of the [other programme] courses are based around working through online units so it offered a different dimension.’ Students also suggested the module’s formative discussion tasks and team project provided real purpose to their co-creation of learning and developed a critical, supportive learning environment. One student writes:

Both the discussion forums and the group project have been very successful in engaging me with other students and I have appreciated the interaction and challenge that this has brought to my ideas.

The group project really is a defining element in helping everyone to contribute and makes it feel more of a learning community.

Reflecting on the two assessments, Owen writes:

I had recently worked with students on extra-curricula projects (Owen and Wasiuk 2021) and experienced the positive, reciprocal impact of co-creation. Therefore, I wanted to implement a more co-created approach in my day-to-day teaching, hoping for a similar experience for both students and staff. I also wanted to try out new assessment formats from those traditionally used on my programme and somewhat challenge the status quo, by providing more inclusive, self-determined assessment opportunities.

I wasn’t sure what to expect from the students’ submissions for the blog, particularly as I was asking students to write about personal experiences on an open platform (Owen et al. 2021). The students valued the opportunity to write about their motivations for studying with some suggesting that the blogs acted like an ice-breaker activity but also offered deeper insights into their peers’ experience and backgrounds.

I had similar concerns around the team project as colleagues tended to steer away from ‘troublesome’ group assessments. However, I felt the team project offered some authenticity as the real world, human-centred context of public health involves people working in multidisciplinary teams to solve complex problems.

The inclusive nature of both assessments provided students with the possibility to ‘configure and customise their own practices’ (Fawns 2019: 140) and continued to be popular assessment activities with the next cohort of students. Subsequently, programme colleagues have also explored use of similar assessment formats on their modules.

## Six Key Practices of Networked Learning and Their Synergies with Curriculum Co-creation

In this section, we examine each of the six key practices described by Hodgson and McConnell (2019), focusing on the synergies with the core elements of curriculum co-creation in theory and practice. Notably, our practical example was not intentionally designed with networked learning theory in mind, yet it reinforces the connections seen in the literature.

### Democracy and Openness in the Educational Process of Learning and Teaching

As in networked learning, curriculum co-creation is widely seen as a process of learning and teaching that promotes democratic decision-making and negotiation about students’ and teachers’ shared values and aims in learning and teaching (Bergmark and Westman 2016; Bron et al. 2016; Deeley and Bovill 2017; Lubicz-Nawrocka 2019b). The blog publication in our example promoted democratic participation of all students and the public nature of the blog allowed it to be shared widely across the programme and the wider public. By contrast, assignments submitted to a closed platform and kept private would usually not have been read by anyone other than the tutor marking them. We recognise that not all learners (or educators) feel comfortable with sharing their work and reflections publicly since it could affect their digital wellbeing (Hibberson, Barrett, and Kelly 2020). However, the open and reflective nature of this assessment received extremely positive feedback from students and staff.

Ryan and Tilbury (2013: 16) describe how ‘[t]he concept of “co-creation” is used to indicate interactions that encourage collaborative and democratic input from students as stakeholders in shaping knowledge practices’. In this respect, academic staff need to demonstrate openness to create learning opportunities that invite students to become co-creators (Bovill and Woolmer 2019; Lubicz-Nawrocka 2019b). The democratic input during co-creation recognises diverse expertise, perspectives, and contributions from students and staff as they work in partnership (Bergmark and Westman 2016; Matthews et al. 2018; Mercer-Mapstone et al. 2018).

Some students in our example raised concerns over how the broad diversity within their teams could lead to disagreements in negotiating the project topic and approach. This is understandable, particularly in teams where everyone is new. However, respect and openness in negotiation are key components of co-creation and partnership (Bovill 2020b) and these were discussed with the students when introducing the team project. Also, some studies suggest that diverse, heterogeneous teams are generally more creative and better at decision-making than homogeneous teams, particularly for high performance tasks (Bowers et al. 2000). Mello and Ruckes (2006: 1038) find that ‘[h]eterogeneous teams generally have an advantage over homogeneous ones in highly uncertain situations and when the stakes in the decisions are high’. It could be argued that an assessed team project is both high stakes (worth 60% of the module’s marks) and an uncertain situation, where students are collaborating with peers and co-creating assessed work for the first time. However, working through these challenges can provide fruitful opportunities for student development, resilience, and transformation (Lubicz-Nawrocka and Bovill 2021).

Early work from Boomer (1992) positions ‘curriculuming’—the process of developing, growing, and adapting a curriculum based on the context and needs of learners and teachers—at the heart of curriculum co-creation and negotiation. Furthermore, Bron et al. (2016: 18) draw on Boomer’s work to argue that curriculum co-creation models important skills and attributes by fostering the ‘development of citizens with democratic abilities to collaborate, negotiate and enquire’. The encouragement of collective inquiry and civic engagement can have profound effects on both learners’ and teachers’ roles and sense of identity (Bergmark and Westman 2016; Lubicz-Nawrocka 2019b). However, it is also important to recognise how democratically negotiated curricula can challenge the status quo of academic cultures, structures, and processes to transform and enhance individuals’ higher education experiences (Bovill 2020b; Lubicz-Nawrocka 2019b).

### Self-determined/Managed Learning and the Freedom to Learn

In our example, students were given the freedom to develop and share their blog posts, promoting their self-determined learning as they creatively crafted text and images to engage readers within and beyond the module participants. Furthermore, working in teams on an authentic problem gave students the ‘opportunity to contribute equally, although not necessarily in the same ways’ (Cook-Sather et al. 2014). A core aspect of curriculum co-creation is how it gives students increased agency when they share responsibility with staff over aspects of decision-making that affect teaching and learning (Bergmark and Westman 2016; Bovill and Bulley 2011; Bovill and Woolmer 2019; Lubicz-Nawrocka 2019b). For example, Ryan and Tilbury (2013: 16) describe how ‘[t]he pedagogical ambitions behind learner empowerment are realised through the use of participatory, transformative and “active” pedagogies’ since they facilitate students’ self-determined learning as well as significant personal and professional development (Lubicz-Nawrocka and Bovill 2021).

Increasing students’ agency and responsibility provides further opportunities for self-determination and freedom to learn within supportive learning environments that promote collaboration and negotiation. Marshalsey and Sclater (2018) find that participatory methods including co-creation can support students’ self-directed learning, creativity, and reflection. Furthermore, Lubicz-Nawrocka (2019a) shows how curriculum co-creation promotes collaboration, creativity, innovation, and enjoyment of learning while balancing risks to help students develop confidence and resilience through authentic learning and teaching experiences.

### Purpose in the Cooperative Process

The blog-based assessment contributed to a sense of learning community and led to deeper student engagement with the module (Owen et al. 2021). Students used the discussion boards to discuss and create connections between their blog posts, the course content, and the formative learning activities. This appeared to foster a sense of purpose and motivation through student collaboration and engagement in the learning process. Along with increased agency while sharing responsibility for co-created curricula, students can also feel a stronger sense of purpose in the cooperative process of learning and teaching.

By its very nature, the democratic, collaborative process of negotiating co-created curricula means that students and staff work towards developing shared aims throughout their course or initiative (Bovill 2020b; Lubicz-Nawrocka 2019b). Student engagement in deep and active learning is often a key benefit for students who negotiate academic content and pedagogy (Backhouse et al. 2019; Billett and Martin 2018; Blau and Shamir-Inbal 2018; Mercer-Mapstone et al. 2017) as well as assessment (Deeley and Bovill 2017; Doyle et al. 2019).

Active learning experiences during co-creation tend to increase students’ motivation to engage in learning (Backhouse et al. 2019; Bergmark and Westman 2016; Fung 2017; Mercer-Mapstone et al. 2017; Owusu-Agyeman and Fourie-Malherbe 2019) as well as staff motivation to engage in teaching (Cook-Sather et al. 2014; Lubicz-Nawrocka 2019b). Furthermore, curriculum co-creation and partnership can promote culturally and personally relevant forms of higher education that are inclusive, which often increases participants’ motivation to engage since they feel a sense of authenticity and purpose in learning and teaching (Keevers 2016; Khasnabis and Reischl 2018; Martin 2018; Martínez-Carrasco 2018; Mercer-Mapstone et al. 2017; Towers and Loynes 2018).

### A Supportive Learning Environment

Curriculum co-creation promotes staff and students’ openness to new ideas and perspectives as well as their agency and cooperation within a vibrant and supportive environment that promotes learning for all involved. In our example, students described feeling part of a supportive learning community fostered by the blog assignment, and some reflected on how reading the personal and emotional experiences of other students helped develop empathy, more so than the common ‘ice-breaker’ activity adopted in many online courses (Owen et al. 2021). The students worked together through purposeful cooperation in a supportive learning environment resulting in what appears to be the beginning of a learning community (Owen et al. 2021).

Curriculum co-creation promotes strong professional relationships between students and between students and staff, which enhance their sense of belonging and community (Bovill and Woolmer 2019; Matthews et al. 2018; Moore-Cherry 2019). These practices can promote an open and supportive dialogue about how to achieve the best outcomes from all engaging in teaching and learning, which helps both students and staff to understand their responsibilities and creates a more equal and reciprocal balance of power between them (Boomer 1992; Kehler, Verwood, and Smith 2017; Moore-Cherry 2019).

There is also a transformative capacity of curriculum co-creation for individuals who are supported to take on non-traditional roles and new identities (Cook-Sather et al. 2014; Hill et al. 2016; Lubicz-Nawrocka and Bovill 2021; Moore-Cherry 2019). This transformation can also extend to staff and institutions that work to develop a culture of partnership and try new approaches to enhance teaching quality (Bovill and Woolmer 2019; Matthews et al. 2018). Curriculum co-creation can promote inclusion of diverse students and staff of different backgrounds and ages, and it can advance notions of democratic values and civic engagement (Bergmark and Westman 2016; Lubicz-Nawrocka 2019b) and social justice (Cook-Sather et al. 2018; Hussain and Wattles 2017).

### Collaborative Assessment of Learning

In the co-creation example in practice, we saw how students informally and collaboratively assessed learning through providing comments and feedback on peers’ blog posts. Furthermore, the scaffolded collaborative assessment of learning was built on during the team projects, where students used peer assessment criteria to evaluate their peers’ individual contributions within their teams before tutors assessed the final projects. Hodgson and McConnell (2019: 51) state: ‘Collaborative self-peer-tutor-assessment processes are central to networked learning: they are a corollary of cooperative learning, and support the cooperative process.’ Like these authors, we agree that students benefit from assessing their own learning and that of peers, with guidance and validation from teachers.

Collaborative, co-created assessment is another area of student-staff partnership which is gaining attention more broadly. Deeley and Bovill (2017: 463) argue that ‘a partnership approach, designed to democratise the assessment process, not only offered students greater agency, but also helped students to enhance their assessment literacy’ as we saw in our example. Hodgson and McConnell (2019) also take the view that collaborative assessment processes are central to networked learning, where negotiation and sensitivity to the learner are crucial for more equal, co-created assessments where the leaner has greater autonomy and agency over their learning.

### Review and Evaluation of the Ongoing Learning Process

In our example, the blogs and outputs from the team projects have both fed into the design and delivery of the following year’s module, not just as examples of previous student work, but also as activities where current students practice peer assessment using the associated marking criteria. This demonstrates opportunities for co-created teaching and learning, both *in* and *of* the curriculum (Bovill and Woolmer 2019). The example demonstrates ongoing co-creation and review of the learning process taking place *in* the curriculum (during the specific module), and an element of module enhancement where the co-created learning and content of current students benefits future students on the next cohort of the module (co-creation *of* the curriculum).

Curriculum co-creation is also seen to develop enhanced meta-cognitive awareness for students (Healey, Flint, and Harrington 2014; Moore-Cherry 2019), since they are becoming more aware of the complexities of engaging in high-impact teaching and learning practices. This helps students to review and evaluate the ongoing learning process, often sharing the pedagogies and practices which they feel they benefit from most while working with staff to assess and enhance learning and teaching. These experiences tend to increase students’ motivation to engage in learning (Backhouse et al. 2019; Bergmark and Westman 2016; Mercer-Mapstone et al. 2017). Other benefits include students’ increased critical thinking skills (Keevers 2016), attainment (Deeley and Bovill 2017), and professional development and employability (Billett and Martin 2018; Dickerson et al. 2016).

Curriculum co-creation is also seen to increase student confidence, self-authorship, and empowerment (Hill et al. 2016; Lubicz-Nawrocka and Bovill 2021; Mercer Mapstone et al. 2017; Moore-Cherry 2019). These increased skills and capacities make students well placed to engage actively with evaluating the learning process for continual improvement, in partnership with staff.

## Discussion

As seen in the example above and throughout the literature, students and staff negotiating curricula and sharing power and responsibility can be challenging. Hierarchies of power in higher education can be deeply embedded and difficult to overcome. However, dialogue and reflection, particularly around roles, identity, and expertise, can help address these concerns and form more equitable partnerships where power is shared between students and staff (Matthews 2017; Owen and Wasiuk 2021). In addition, curriculum co-creation has been seen as a good practice in higher education (Bovill 2013) that can be transformational for students (Lubicz-Nawrocka and Bovill 2021). This transformation has been seen to benefit not only individuals within modules’ closed learning communities but also the wider public through open educational practices (Cook-Sather et al. 2014; Lubicz-Nawrocka 2019b; Bovill 2020a; Owen and Wasiuk 2021).

Chickering and Gamson’s ‘Seven Principles for Good Practice in Undergraduate Education’ (1987: 3) have been used for many years before the advent of networked learning and curriculum co-creation. These principles include student-staff interaction, reciprocity in peer learning, active learning, prompt feedback, time on task, high expectations of students, and respecting students’ diverse talents and ways of working. Some of these principles also connect closely to Hodgson and McConnell’s (2019) six practices of networked learning. In writing about curriculum co-creation, Bovill (2013: 472) states: ‘There is no guarantee that any particular co-created curricular initiative will meet all of Chickering and Gamson’s seven principles of good practice, but the fundamental aims of students and staff co-creating curricula are certainly broadly consistent with these principles of good practice.’ Lubicz-Nawrocka (2020) also found that each of these seven principles of good learning appear to be intertwined within co-creation of the curriculum, which takes many of the principles to new levels of high-quality learning and teaching compared to their enactment through other forms of student learning and engagement.

Lubicz-Nawrocka (2020) highlights various ways in which curriculum co-creation can advance students’ personal and professional development as well as contemporary notions of teaching excellence. In particular, co-creation fosters teaching excellence by developing students’ agency, critical thinking skills, and employability skills as well as moral excellence by placing students at the heart of the learning experience. Moral excellence is conceptualised by Kreber (2007: 237) as ‘to do what is good’ and, first and foremost, for staff ‘to do what is in the best interest of learners’. This is also seen in Hodgson and McConnell’s (2019: 45) first principle of networked learning, where ‘[t]he focus is on learning which has a perceived value to the learners’. There are also many ways in which networked learning and curriculum co-creation promote high-impact educational practices that facilitate student engagement and academic success, such as how they facilitate participation in common intellectual experiences, learning communities, and collaborative assignments and projects (Kuh 2010).

## Conclusion

Learning and teaching during the Covid-19 pandemic has helped reinforce the interconnected nature of postdigital education that recognises the effective environments that foster learning not only for individual students but also collective learning for students and staff (Jandrić et al. 2020, 2021). The values that serve as a foundation for our learning communities help foster embodied and personally relevant learning based on trust, respect, and reciprocity as we open up opportunities for students to actively co-create aspects of their educational journeys.

We demonstrate analytical generalization (Smith 2018) by showing the connections between practices and theories of curriculum co-creation and networked learning. Recognising how these two approaches are both grounded in critical inquiry, we hope we have also provided provocative generalizability (Smith 2018) whereby readers may be inspired to think in new ways about the possibilities that curriculum co-creation could offer in postdigital contexts.

The theories and practices underpinning each appear to have arisen in different realms of the higher education literature. However, this article shows the connections between curriculum co-creation and the six practices of networked learning set out by Hodgson and McConnell (2019). These approaches to higher education can enhance openness, self-determination, purpose, collaboration, support, reflection, and evaluation. Equally, they can foster creativity and authentic learning through both the process of engaging collaboratively with diverse students and developing resources—such as blogs—that can benefit the wider public, especially when engaging in assessments that advance open education.

Networked learning and curriculum co-creation both capture many elements of high-impact, excellent learning and teaching that have the capacity to be transformative for students as they engage in postdigital education. Postdigital thinking and dialogue during curriculum co-creation fosters collective intelligence within supportive learning environments by respecting students’ contributions as they share responsibility for the cross-fertilisation of ideas and learning. These experiences give students agency, which can drive their motivation to engage in active learning and develop attributes such as democratic engagement which should benefit students as well as staff during the pandemic and beyond.

## Footnotes

1 See https://www.manchester.ac.uk/study/masters/courses/list/06002/mph-public-health-web-based-learning/. Accessed 8 March 2022.
